# Lactylation Modification as a Promoter of Bladder Cancer: Insights from Multi-Omics Analysis

**DOI:** 10.3390/cimb46110766

**Published:** 2024-11-13

**Authors:** Yipeng He, Lingyan Xiang, Jingping Yuan, Honglin Yan

**Affiliations:** 1 Department of Pathology, Renmin Hospital of Wuhan University, Wuhan 430060, China; heyipeng@whu.edu.cn (Y.H.); 2023283020213@whu.edu.cn (L.X.); yuanjingping@whu.edu.cn (J.Y.); 2 The First Clinical College, Wuhan University, Wuhan 430060, China

**Keywords:** multi-omics analysis, lactylation modification, bladder cancer

## Abstract

Bladder cancer (BLAC) is a malignant tumor with high morbidity and mortality. The establishment of a prognostic model for BLAC is of great significance for clinical prognosis prediction and treatment guidance. Lactylation modification is a newly discovered post-transcriptional modification of proteins, which is closely related to the occurrence and development of tumors. Multiple omics data of BLAC were obtained from the GEO database and TCGA database. The Lasso algorithm was used to establish a prognostic model related to lactylation modification, and its predictive ability was tested with a validation cohort. Functional enrichment analysis, tumor microenvironment analysis, and treatment response evaluation were performed on the high- and low-risk groups. Single-cell and spatial transcriptome data were used to analyze the distribution characteristics of model genes and their changes during epithelial carcinogenesis. A prognostic model consisting of 12 genes was constructed. The survival rate of the high-risk group was significantly lower than that of the low-risk group. The multiple ROC curve showed that the prediction efficiency of the model was higher than that of the traditional clinical tumor grading. Functional enrichment analysis showed that glycolysis and hypoxia pathways were significantly upregulated in the high-risk group. The high-risk group was more sensitive to most first-line chemotherapy drugs, while the low-risk group had a better response to immunotherapy. Single-cell sequencing analysis revealed the dynamic changes of model genes during the transition of epithelial cells to squamous-differentiated cells. Spatial transcriptome analysis showed the spatial distribution characteristics of the model genes. The lactylation-related models have a satisfactory predictive ability and the potential to guide the clinical treatment of BLAC. This model has significant biological implications at the single-cell level as well as at the spatial level.

## 1. Introduction

Bladder cancer (BLAC) is the fourth most common malignant tumor worldwide [1]. This malignant disease is increasing at a rate of approximately 500,000 new cases and 200,000 deaths per year [2]. BLAC can be divided into muscle-invasive BLAC and non-muscle-invasive BLAC. For the former group, the 5-year survival rate is less than 40%, and nearly half of the patients will develop distant metastasis within 3 years after surgery [1,3,4]. Although the prognosis of non-muscle-invasive BLAC is relatively better, it is one of the most costly tumors to treat, owing to the frequent need for cystoscopy and transurethral resection of the bladder tumor (TURBt) [5]. Therefore, it is of great significance to study the occurrence and development of BLAC and to predict the prognosis of patients and guide treatment.

Lactylation modification is a novel post-translational modification of proteins. Zhang et al. first discovered and described histone lactylation modifications in humans and mice by using high-performance liquid chromatography (HPLC)–tandem mass spectrometry (MS/MS), and since then, lactate has also been shown to play an important role in the field of epigenetics [6]. As research on this specific protein modification continues to advance, lactylation modification has been demonstrated to play a decisive role in a variety of key biological processes, such as biochemical pathways related to glycolysis [7], neurodevelopmental processes [8], immunosuppression [9], tumor promotion [10], and macrophage polarization [6,11].

In the tumor microenvironment, tumor cells metabolically reprogram the entire microenvironment through autocrine and paracrine mechanisms [12]. This metabolic reprogramming causes the entire tumor microenvironment to become locally hypoxic and lactate rich, a phenomenon known as the Warburg effect [13]. Recently, increasing evidence has shown that an increase in lactate level in the tumor microenvironment can directly lead to an increase in intracellular protein lactylation level in the environment [14,15,16,17]. This increased level of lactylation in turn regulates transcription, exacerbating the malignant behavior of tumor cells [18,19]. On the other hand, the function of immune infiltrating cells is inhibited [6,17,20]. In conclusion, the upregulation of protein lactylation modification is often a favorable factor for the development and progression of tumor cells.

Most of the current studies have found that lysine 18 of histone H3 (H3K18) is extensively modified in tumors. In BLAC, histone H3 lysine 18 lactylation (H3K18la) has been shown to confer enhanced resistance to cisplatin in a cisplatin-resistant subpopulation of epithelial cells [21]. Another study demonstrated that CircXRN2 could exert anti-tumor effect by inhibiting H3K18la [10]. PFK-1 can also inhibit the progression of BLAC by suppressing H3K18la [22]. In conclusion, lactylation modification plays a key role in BLAC. However, a prognostic model for BLAC based on lactylation modification is not available.

In this study, we first established a novel prognostic model for BLAC based on the lactylation modification-related genes by using the least absolute shrinkage and selection operator (Lasso) algorithm. External data were introduced to validate the strong predictive power of this prognostic model. Functional enrichment analysis showed that glycolysis and hypoxia pathways were significantly upregulated in the high-risk group. In addition, the differences in tumor microenvironment and treatment response between high- and low-risk groups were carefully compared. Moreover, this study employed cutting-edge transcriptomic methodologies, such as single-cell transcriptomics and spatial transcriptomics, to delve deeper into the prognostic models. It is noteworthy that this paper revealed the alterations of model genes as bladder cells progress from urothelial to squamous epithelial cancer at the single-cell level. Furthermore, spatial transcriptomic sequencing data uncovered the spatial distribution characteristics of model genes. In conclusion, this study established a new prognostic model based on lactylation modification, which is of great significance for clinical diagnosis, prognosis evaluation and treatment guidance.

## 2. Material and Methods

### 2.1. Data Retrieval and Processing

The mRNA transcriptome profiles and corresponding clinical information for the TCGA-BLAC project were retrieved from The Cancer Genome Atlas (TCGA) database (https://portal.gdc.cancer.gov/, accessed on 1 October 2023). Similarly, the data for 308 samples of the GSE32894 project were sourced from the Gene Expression Omnibus (GEO) database (https://www.ncbi.nlm.nih.gov/geo/, accessed on 1 October 2023). Both the TCGA-BLAC project and GSE32894 project datasets were integrated to form the training cohort of the model. The external validation cohort was composed of the samples from the GSE13507 project within the GEO database. Batch effects were corrected using the SVA package in R. Single-cell sequencing data and spatial transcriptome data were obtained from GSE135337 and GSE182211 projects of GEO database, respectively.

Seven lactylation-modified writing and erasing enzymes were reported in previously published studies [6,23]. Wan et al. identified a series of lactylation modification sites in human cell lines [24]. Based on the above findings, 332 genes related to lactylation modification were obtained.

### 2.2. Identifying Prognostic Lactylation-Related Genes

Transcriptome data were analyzed using the “limma” package. The guidelines for the identification of DEGs are a false discovery rate (FDR) < 0.05 and |log2 FC| ≥ 1.25. Differential gene expression heatmaps were produced using the “pheatmap” package. Univariate cox analysis was used to analyze the transcriptome information and survival information to obtain prognostic genes. A Venn diagram was generated using the “venn” package. The “survival” package was used to generate the forest plot of genes related to lactylation modification. The PPI network was constructed using the string website (https://string-db.org/cgi/input.pl, accessed on 1 October 2023), and Cytoscape was used to optimize the network picture.

### 2.3. Functional Enrichment Analysis

Using the R package “limma”, a Wilcoxon test was performed with a threshold of FDR < 0.05 and |log2 FC| ≥ 1, in order to identify differentially expressed genes between the two groups. Further, gene ontology (GO) and Kyoto Encyclopedia of Genes and Genomes (KEGG) enrichment analyses were conducted utilizing the R packages “clusterProfiler”, “org.Hs.eg.db”, “stringi” and “ggplot2” to annotate biological functions and pathways significantly associated with the differentially expressed genes. Gene Set Enrichment Analysis (GSEA) was carried out using the GSEA website (https://www.gsea-msigdb.org/gsea/, accessed on 1 October 2023) to investigate the enrichment of pathways such as glycolysis, among others.

### 2.4. Construction of Lactylation-Related Score Model

A gene model was developed leveraging the “glmnet” and “survival” R packages. To pinpoint an appropriate collection of potential genes as prognostic biomarkers, Lasso regression was applied. Lasso regression was chosen for its effectiveness in handling high-dimensional data and its ability to perform variable selection, allowing us to identify the most relevant genes while minimizing overfitting. This process involved generating the most favorable penalty parameter λ to ascertain the weight coefficients for the genes within a risk score equation. A risk score model was built using the training dataset as follows:

$$Riskscore=\sum_{i=1}^{N}\left(exp\times coef\right)$$

*N* is the number of model genes; *exp* represents the expression value of the gene; *coef* is the coefficient for each gene. Patients in the training set were divided into two groups, and Kaplan–Meier analysis was performed to assess the difference in overall survival time (OS) between the two groups. Kaplan–Meier analysis was selected because it provides a clear and informative visual representation of survival probabilities over time, which is crucial for understanding the prognostic implications of the identified genes. Receiver operating characteristic (ROC) curves were generated using the R package (R 4.3.2) “timeROC” to assess the sensitivity and specificity of the risk model. ROC analysis was employed to evaluate the diagnostic accuracy of our risk score, helping to determine the optimal threshold for patient stratification. An external validation cohort was used to validate the model.

### 2.5. Mutation Analysis

Mutation data from the TCGA-BLAC project were downloaded from the TCGA database. The overall picture of the mutated genes for BLAC was obtained by using the “plotmafSummary” function in the “maftools” package. The “oncoplot” function was used to plot the twelve model genes.

### 2.6. Tumor Immune Microenvironment Analysis

The ssgsea algorithm was used to analyze the immune cell infiltration and immune function in the tumor microenvironment. The “limma”, “GSVA”, and “GSEABase” packages were used for analysis. The “reshape” and “ggpubr” packages were used for subsequent analysis and drawing. Heatmaps of the relationship between model genes, risk scores, and the immune microenvironment were produced by “pheatmap”. The “estimate” package was used for overall assessment of the tumor microenvironment and tumor purity analysis.

### 2.7. Assessment of Treatment Response

Drug sensitivity assessment was implemented through the “pRRophetic” package, and the basic idea relied on the construction of statistical models that first use gene expression data from large cancer cell line databases to determine the IC50 sensitivity threshold of different drugs to the cell line. The model is then applied to gene-expression profiling data from tumor samples to predict their likely susceptibility to specific drugs. When the results are shown, boxplots are used to compare the IC50 distribution of each therapeutic drug with significant differences between the experimental and control groups. The response to immunotherapy was assessed with access to Tumor Immune Dysfunction and Exclusion (TIDE), an online tool available from Harvard University (http://tide.dfci.harvard.edu/, accessed on 1 October 2023). The TIDE framework quantitatively assesses the possibility of tumor evasion from an immune system attack based on the gene expression characteristics of tumor samples.

### 2.8. Single-Cell Analysis

Single-cell analysis was mainly achieved through the “seurat” package. After quality control, data standardization, and unsupervised clustering, the umap method was used to reduce the dimension of the analysis. Marker genes of cell subsets were examined using the “findallmarkers” function. The “SingleR” package was used to assist the cell annotation work.

Specifically, it is worth mentioning the annotation of the squamous differentiated cell cluster. This cell cluster was classified as epithelial cells in the SingleR annotation results. However, in the UMAP plot, this cell cluster is located relatively far from the actual epithelial cell cluster, indicating a certain degree of differentiation distance from epithelial cells. Moreover, only this cell cluster highly expresses S100A7 and S100A8. Through the CellMarker website (http://biocc.hrbmu.edu.cn/CellMarker/, accessed on 1 October 2023), we found that these two markers have been reported as markers of squamous cell carcinoma [25,26]. Therefore, based on the combination of machine and manual annotation, this cell cluster was ultimately annotated as squamous differentiated epithelial cancer cells.

Cell communication analysis was performed using the “cellchat” package and the “tidyverse” package. Squamous differentiated and epithelial cell types were selected for the pseudotime analysis, which was implemented on the basis of the “monocle” package.

### 2.9. Spatial Transcriptome Analysis

Spatial transcriptome analysis was conducted based on the “seurat”, “dplyr”, “patchwork” and “ggplot2” packages. The “Load10X_Spatial” function was used to read spatial transcriptome data. The spatial transcriptome data were normalized using the SCTransform algorithm. The expression distribution map of genes in space was completed using the “SpatialFeaturePlot” function.

## 3. Results

### 3.1. Identification of Prognostic Lactylation-Related Genes in BLAC

**Figure 1:**
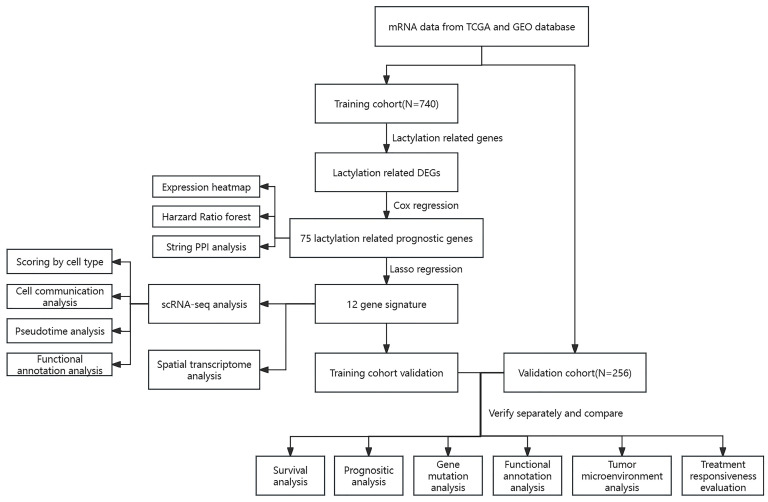
Study workflow diagram.

**Figure 2:**
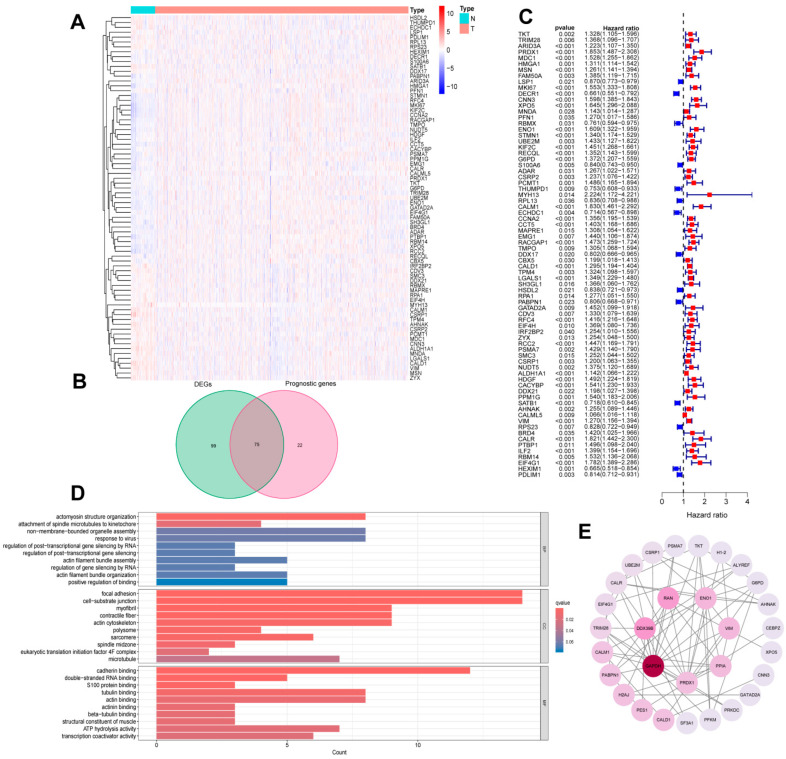
Identification of prognostic lactylation-related genes in BLAC. (**A**) A heatmap presenting the expression levels of the lactylation-related DEGs. (**B**) A Venn diagram showing the quantities of the prognostic genes and DEGs. (**C**) A forest plot of prognostic lactylation-related DEGs. (**D**) A bar plot of GO analysis based on DEGS. (**E**) A PPI network showing known and predicted interactions of proteins and genes among the prognostic lactylation-related DEGs.

Figure 1 shows the overall flow of the article. Based on transcriptome expression data derived from BLAC and normal bladder tissue samples, the expression information of lactylation-related genes was selectively extracted for further analysis. Through differential analysis, 174 differentially expressed genes (DEGs) were identified in BLAC. A heatmap was constructed to visualize the expression patterns of these differential genes across tumor and normal groups (Figure 2A). Survival analysis of the transcriptomic data, informed by survival information from BLAC patients, resulted in the identification of 97 genes significantly associated with patient prognosis. As depicted in a Venn diagram (Figure 2B), an intersection was identified between the set of DEGs and the set of prognostic genes, resulting in the extraction of 75 lactylation-associated genes common to both analyses. A forest plot was then generated to display the hazard ratios of these 75 intersecting genes (Figure 2C). Gene Ontology (GO) enrichment analysis revealed that these lactylation-modified genes were significantly enriched in cellular components related to cell junction structures and involved in binding processes with numerous other biomolecules, such as cadherins (Figure 2D). Moreover, Protein–Protein Interaction (PPI) network analysis indicated that these genes exhibit a complex and highly interconnected relationship (Figure 2E).

### 3.2. Construction of Lactylation Risk Model

**Figure 3:**
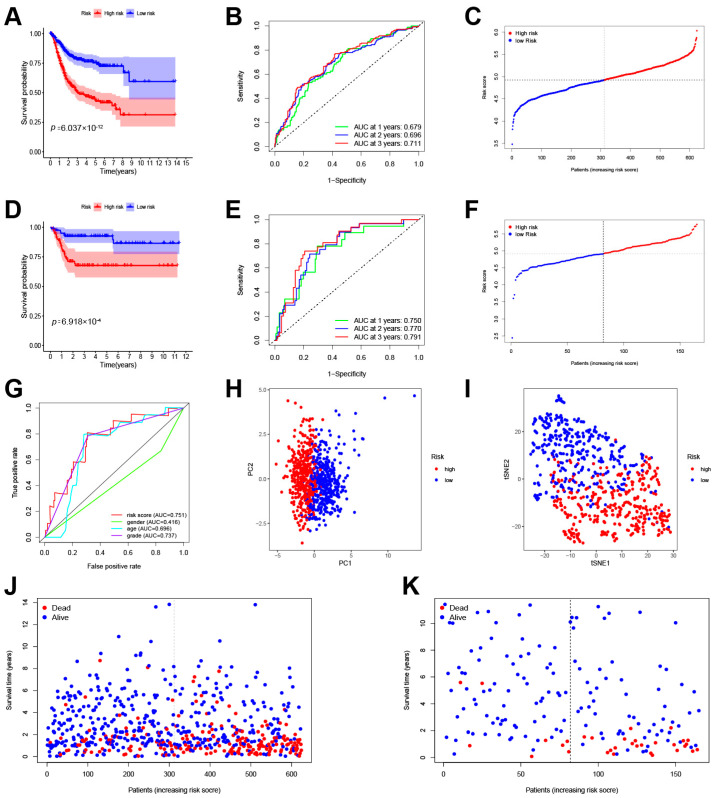
The construction of the prognostic signature. (**A**) Kaplan–Meier survival curves of the HCC overall survival in the training cohort. (**B**) ROC curves showing the predictive efficacy of the prognostic signature in the training cohort. (**C**) The distribution of the risk score in the training cohort. (**D**) Kaplan–Meier survival curves of the HCC overall survival in the validation cohort. (**E**) ROC curves showing the predictive efficacy of the prognostic signature in the validation cohort. (**F**) The distribution of the risk score in the validation cohort. (**G**) Multi-ROC curves showing the predictive efficacy of different clinical traits. (**H**) PCA of all patients in the training cohort and validation cohort. (**I**) T-SNE analysis of all patients in the training cohort and validation cohort. (**J**) The distribution of survival status with an increasing risk score in the training cohort. (**K**) The distribution of survival status with an increasing risk score in the validation cohort.

**Figure 4:**
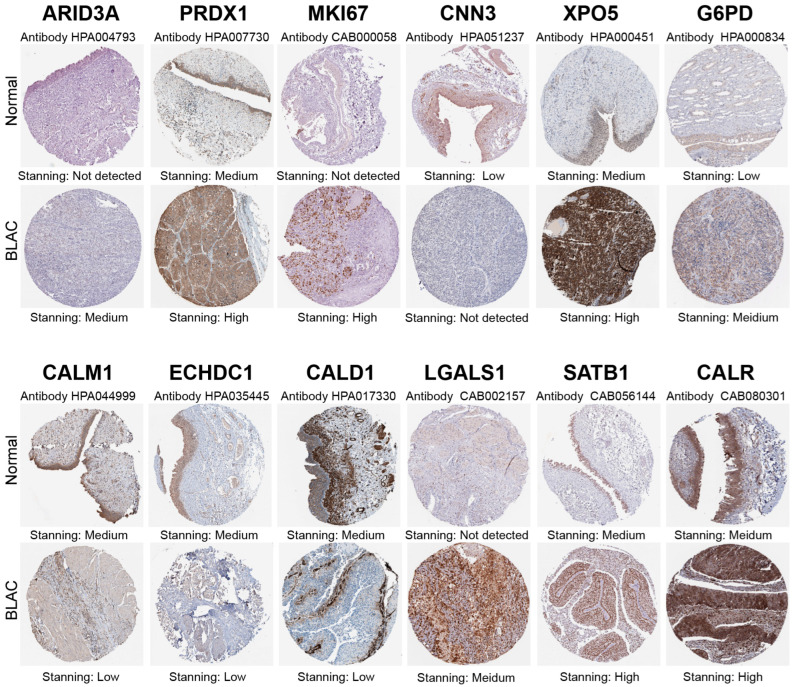
Validation of immunohistochemical staining from the HPA database. The differential expression of the twelve model genes in bladder cancer compared to adjacent normal tissue is consistent with the results from RNA-seq analysis.

Lasso–Cox regression analysis was conducted on the training cohort composed of TCGA-BLAC and GSE32894 projects, resulting in a risk scoring model comprising 12 genes. The score formula for the prognostic model is as follows (each gene symbol represents its mRNA expression level): score = 0.013329729 × ARID3A + 0.060866365 × PRDX1 + 0.001150845 × MKI67 + 0.11019392 × CNN3 + 0.061662743 × XPO5 + 0.165375341408274 × G6PD + 0.0918858789393128×CALM1 − 0.00915801596702691 × ECHDC1 + 0.00813049916598936 × CALD1 + 0.0814907938842537 × LGALS1 − 0.0677031902775691 × SATB1 + 0.057526713 × CALR. Based on this model, the training cohort was stratified into high-risk and low-risk groups. The Kaplan–Meier survival curve (Figure 3A) revealed significantly lower survival rates for the high-risk group compared to the low-risk group (*p* = 6.036 × 10^−12^). ROC analysis was performed to evaluate the predictive capability of the model (Figure 3B). The scores of the training cohort are shown in Figure 3C. Subsequently, a validation cohort composed of the GSE13507 program was used to assess the overall predictive power of the model. As anticipated, the model demonstrated robust predictive capacity in the validation cohort, with significantly decreased survival rates observed in the high-risk group relative to the low-risk group (*p* = 6.918 × 10^−4^) (Figure 3D). The ROC curves showed area under the curve (AUC) values of 0.750, 0.770, and 0.791 at one year, three years, and five years, respectively (Figure 3E). A scatter plot was plotted to depict the scores in the validation group (Figure 3F). Moreover, multiple ROC curves were constructed based on the risk scores from the validation cohort juxtaposed against other established clinical characteristics. These multi-ROC analyses indicated that the risk score had the highest AUC among all clinical traits, outperforming the traditional clinical grading system (Figure 3G), thus further substantiating the satisfactory predictive ability of the lactylation-related gene prognosis model. Principal Component Analysis (PCA) and t-Distributed Stochastic Neighbor Embedding (t-SNE) dimensionality reduction confirmed that the model effectively segregated samples into two distinct categories (Figure 3H,I). Overall, there is a consistent trend where higher risk scores in the model correspond to shorter survival times in patients (Figure 3J,K). In conclusion, the risk scoring model derived from the Lasso–Cox regression has shown compelling prognostic value across both the training and validation cohorts, providing a promising tool for predicting patient outcomes. Immunohistochemical staining confirmed that the differential expression of the twelve model genes is consistent with the results described above (Figure 4).

### 3.3. The Prognostic Value of the Lactylation Risk Model

**Figure 5:**
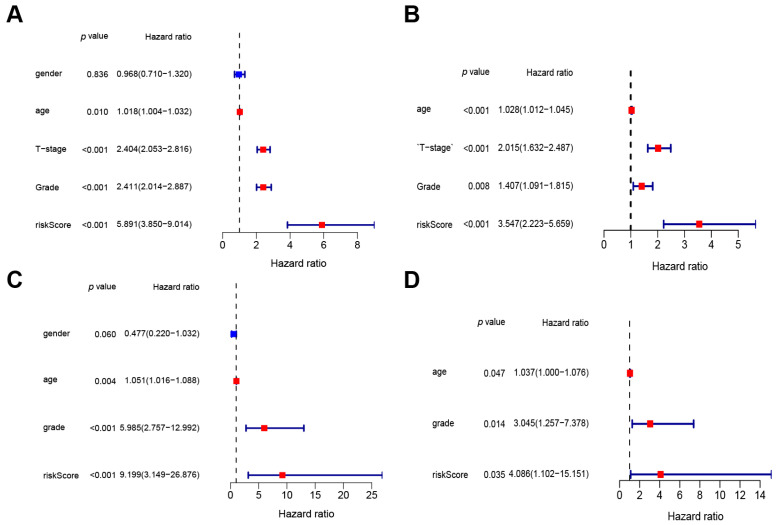
Univariate and multivariate independent prognostic analyses. (**A**) Univariate independent prognostic analysis in training cohort. (**B**) Multivariate independent prognostic analysis in training cohort. (**C**) Univariate independent prognostic analysis in validation cohort. (**D**) Multivariate independent prognostic analysis in validation cohort.

Independent prognostic analysis was performed on combined transcriptomic and clinical information to assess the predictive value of the risk model related to lactylation-associated genes in the prognosis of BLAC. In the training set, univariate independent analysis identified T stage, grade, and risk score as significant risk factors for BLAC (Figure 5A). Subsequent multivariate independent prognostic analysis revealed that the risk score remained a statistically significant risk factor for BLAC (Figure 5B). In the validation cohort, both univariate and multivariate independent prognostic evaluations consistently showed that the risk score functioned as a significant risk determinant for BLAC (Figure 5C,D). Collectively, these results affirm that the risk score derived from the lactylation-related gene risk model serves as an independent risk factor for BLAC.

### 3.4. Mutation Analysis and Functional Enrichment Analysis

**Figure 6:**
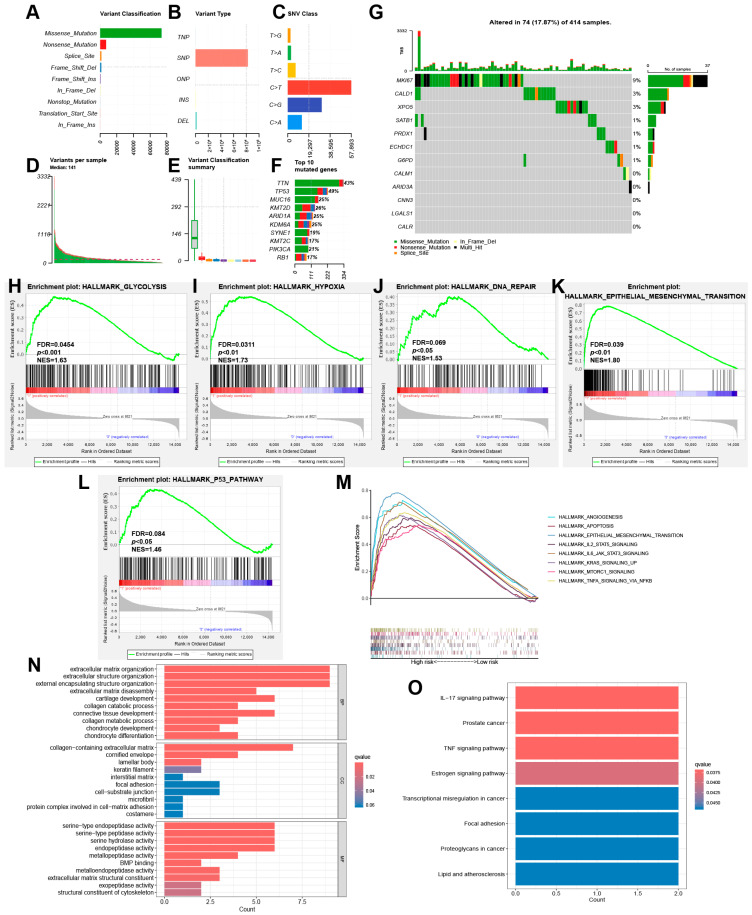
Mutation analysis and functional enrichment analysis. (**A**) Variant classification in BLAC. (**B**) Variant type in BLAC. (**C**) Single-nucleotide variation class in BLAC. (**D**) Variants per sample in BLAC. (**E**) Variant classification summary in BLAC. (**F**) Top 10 mutated genes in BLAC. (**G**) Waterfall plot of mutation frequencies of 12 model genes. (**H**–**M**) Highly expressed GSEA pathway in high-risk group. (**N**) GO analysis based on differentially expressed genes between high- and low-risk groups. (**O**) KEGG analysis based on differentially expressed genes between high- and low-risk groups.

Tumor gene mutation analysis was performed to investigate the mutation status of risk model genes in BLAC patients. The overall profile of gene mutations in BLAC is first shown in Figure 6A–F. Among all mutation types, missense mutations and single-nucleotide mutations had the highest frequency. TTN, TP53 and MUC16 are the three most frequently mutated genes in BLAC. A mutation waterfall plot was generated to display the mutations of the risk model genes in BLAC (Figure 6G). In addition, functional and pathway enrichment analysis was performed on the high- and low-risk groups divided by the risk model. The results of GSEA enrichment analysis showed that glycolysis and hypoxia pathways were significantly enriched in the high-risk group (Figure 6H,I). This is consistent with our expectation because the level of lactylation modification is closely related to the anaerobic glycolysis pathway in the tumor microenvironment. Moreover, DNA repair, the epithelial–mesenchymal transition, and the p53 pathway are also enriched in the high-risk group (Figure 6J–L). In addition, cellular processes such as the epithelial–mesenchymal transition and signaling pathways such as the KRAS pathway were also enriched in the high-risk group (Figure 6M). GO analysis showed that many processes related to extracellular matrix construction and degradation were enriched, suggesting the possibility of tumor cell metastasis and enhanced matrix remodeling capacity in the high-risk group (Figure 6N). KEGG analysis demonstrated the involvement of numerous signaling pathways (Figure 6O).

### 3.5. Tumor Immune Microenvironment Analysis

**Figure 7:**
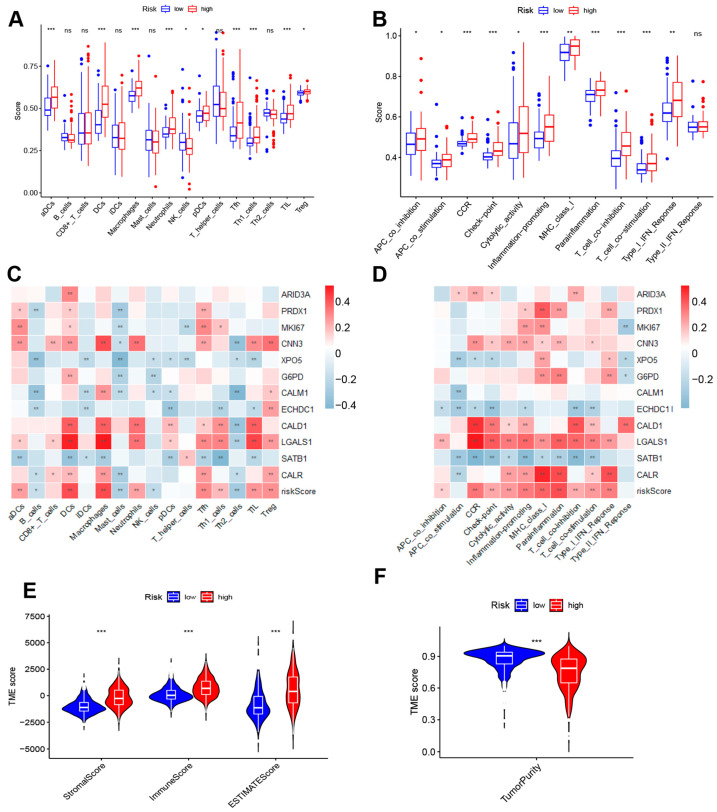
Tumor immune microenvironment analysis (**A**,**B**) Comparison of immune infiltration and immune function between high- and low-risk groups. (**C**) Heatmap of relationship between 12 model genes, risk scores, and immune infiltration (* *p* < 0.05, ** *p* < 0.01, *** *p* < 0.001). (**D**) Heatmap of relationship between 12 model genes, risk scores, and immune function (* *p* < 0.05, ** *p* < 0.01, *** *p* < 0.001). (**E**) StromalScore, ImmuneScore, and ESTIMATEScore of high- and low-risk groups based on ESTIMATE analysis. (**F**) Tumor purity in high- and low-risk groups based on ESTIMATE analysis.

The immune microenvironment is an important part of the tumor microenvironment, a key factor influencing the onset, progression, and prognosis of tumors, as well as an important guiding indicator for the development of treatment strategies. In order to elucidate the differences in the immune microenvironment between the high-risk and low-risk groups as defined by the risk model, a comprehensive depiction of cell infiltration and cellular functions within the microenvironment is initially presented (Figure 7A,B). In the high-risk group, the infiltration scores of dendritic cells (DCs), neutrophils, Th1 cells, and Tregs are relatively higher, indicating a higher level of infiltration of these cells in the high-risk group. In contrast, B cells and NK cells show relatively higher infiltration levels in the low-risk group. Such distribution differences may reflect distinct immune microenvironment characteristics between high- and low-risk patients. Two heatmaps were specifically constructed to elucidate the intricate and significant relationship between the risk model genes, risk scores, and the immune microenvironment, indicating that the twelve lactylation modification-associated genes, along with the risk scores, have profound correlations with various immune cell infiltrations and immune functions (Figure 7C,D). Furthermore, we utilized the ESTIMATE algorithm to analyze the tumor microenvironment from a different perspective, which revealed that the stromal score, immune score, and ESTIMATE score were significantly higher in the high-risk group compared to the low-risk group (Figure 7E). Additionally, the tumor purity in the high-risk group was found to be lower than that in the low-risk group (Figure 7F).

### 3.6. Treatment Responsiveness Evaluation

**Figure 8:**
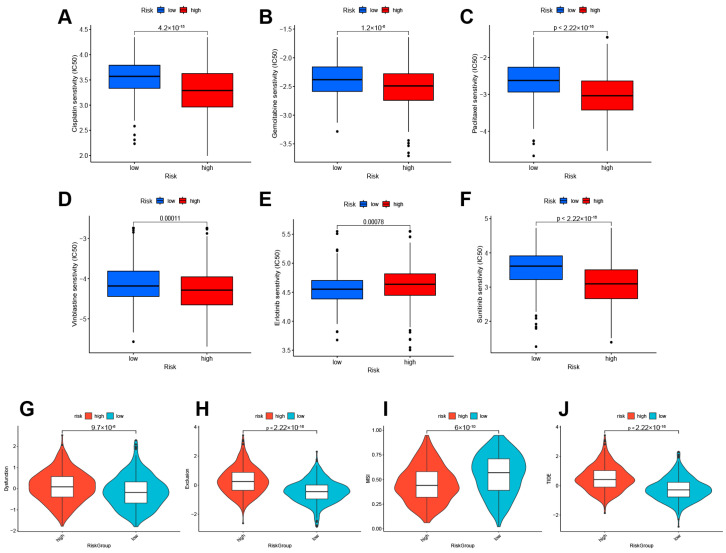
Treatment responsiveness evaluation. Predicted responsiveness to chemical drugs cisplatin (**A**), gemcitabine (**B**), paclitaxel (**C**), and vinblastine (**D**) in BLAC. Responsiveness to targeted drugs erlotinib (**E**) and sunitinib (**F**) in BLAC. (**G**–**J**). Tide scores in high- and low-risk groups.

Pharmacogenomic sensitivity analysis was conducted to assess the predictive capacity of lactylation modification-related gene models in guiding treatment strategies. Among all drugs with statistically significant findings, frontline chemotherapeutic agents for BLAC were of particular interest, such as cisplatin, gemcitabine, paclitaxel, and others. The results indicated that high-risk patients demonstrated increased sensitivity to first-line chemotherapy drugs. Specifically, for cisplatin (Figure 8A), gemcitabine (Figure 8B), paclitaxel (Figure 8C), and vinblastine (Figure 8D), the high-risk group exhibited stronger drug sensitivity, suggesting that this subset of patients is more likely to benefit from these chemotherapy regimens. Conversely, the low-risk group showed higher sensitivity to erlotinib (Figure 8E), while the high-risk group was more sensitive to sunitinib (Figure 8F). Furthermore, Tumor Immune Dysfunction and Exclusion (TIDE) analysis was performed to assess the sensitivity of high- and low-risk groups to immunotherapy. TIDE analysis is a widely used method to predict the response of patients to immunotherapy. A high TIDE score implies a higher probability of benefiting from immunotherapy. The TIDE scores were found to be higher in the high-risk group than in the low-risk group (Figure 8G,H), signifying a stronger capacity for immune escape in the high-risk group. Moreover, when compared to the low-risk group, the high-risk group exhibited lower Microsatellite Instability (MSI) scores (Figure 8I) and correspondingly higher TIDE scores (Figure 8J), indicating that patients in the high-risk group are likely to respond less effectively to immunotherapy treatments.

### 3.7. Single-Cell RNA-Seq Profiling in BLAC

**Figure 9:**
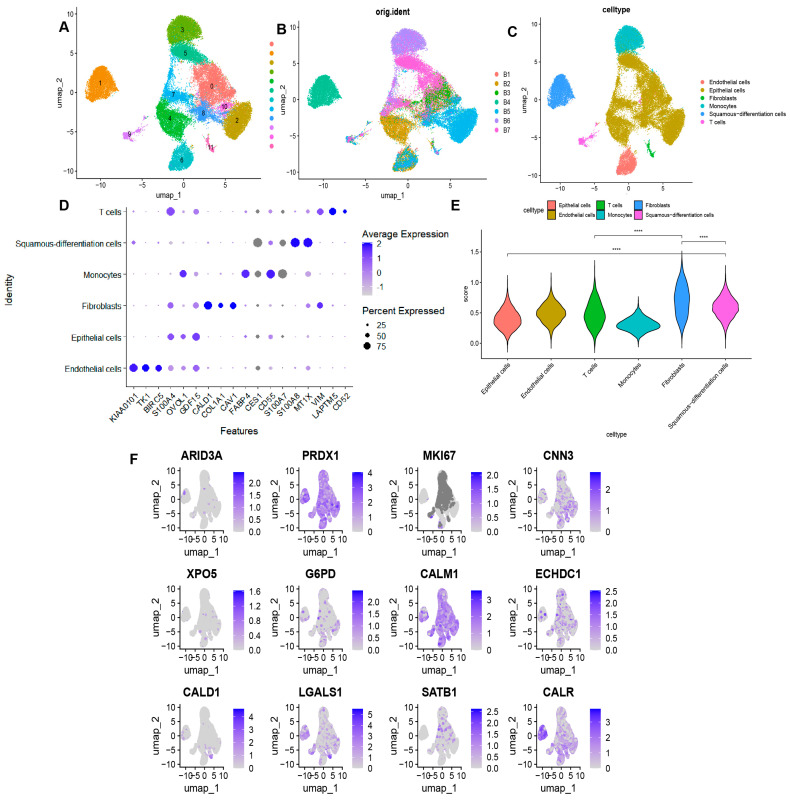
Single-cell RNA-seq profiling in BLAC. (**A**) Umap for dimensionality reduction of data derived from BLAC tissues. (**B**) Umap showing integration of 7 single cell tissue samples. (**C**) Annotation of each cell cluster by singleR and manual annotation. (**D**) Bubble map showing expression of marker genes in each cell type. (**E**) Scores for each cell population based on signature of 12 genes. **** *p* < 0.0001. (**F**) Expression of 12 model genes in each cell type.

Single-cell sequencing offers a more granular perspective on the transcriptomic information within tumors. To investigate the biological significance of lactylation modification-related prognostic models at the single-cell level, we analyzed seven BLAC single-cell samples (comprising a total of 36,788 cells) from the GSE135337 project. Firstly, UMAP dimensionality reduction clustering was carried out on the obtained transcriptome data (Figure 9A). The merged representation of the seven BLAC samples is shown in Figure 9B. Based on the annotations in the original literature in combination with SingleR annotation, we identified and annotated six distinct cell types: endothelial cells, epithelial cells, fibroblasts, monocytes, squamous differentiated cells (a type of malignant BLAC cell), and T cells (Figure 9C). A bubble plot was created to visualize marker genes specific to each cell type (Figure 9D). Subsequently, the risk model was applied to assign scores to each cell type (Figure 9E), revealing that fibroblasts had the highest risk scores. Moreover, squamous differentiated cells displayed higher risk scores compared to epithelial cells. The expression profiles of the twelve risk model genes across the various cell types are presented in Figure 9F.

### 3.8. Cell Communication Analysis, Pseudotime Analysis, and Functional Enrichment Analysis

**Figure 10:**
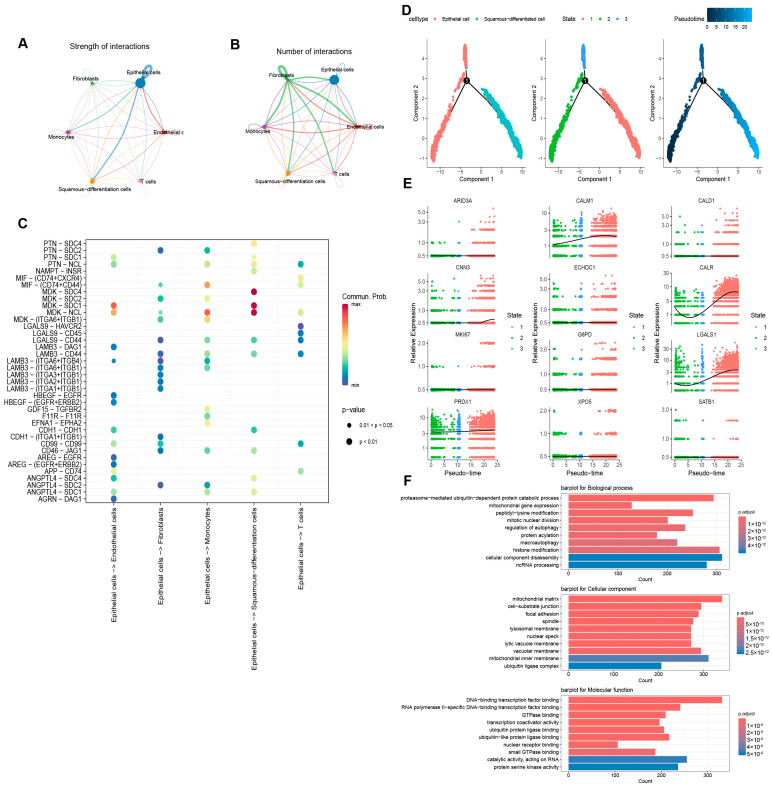
Cell communication analysis, pseudotime analysis, and functional enrichment analysis. (**A**,**B**) The amount and intensity of communication between cell types. (**C**) Receptor–ligand interactions between epithelial cells and other cells. (**D**) Pseudotime analysis of differentiation from epithelial cells to squamous cells. (**E**) Changes in the expression of the 12 model genes as epithelial cells differentiated into squamous cells. (**F**) GO analysis based on differentially expressed genes in squamous differentiated cells and epithelial cells.

Cell communication analysis was performed for diverse cell types to elucidate the intercellular communication in BLAC. In terms of the communication intensity, epithelial cells exhibit the strongest communication with themselves and other cell types (Figure 10A). In regard to the quantity of communications, fibroblasts demonstrate the most extensive exchanges with themselves and other cells (Figure 10B). A bubble plot was constructed to illustrate the receptor–ligand pairs involved in the communication between epithelial cells and other cell types (Figure 10C). In addition, considering that squamous differentiated cells are highly malignant epithelial cells, we performed a pseudotime analysis of these cells and epithelial cells to explore the genetic alterations during this differentiation (Figure 10D). Figure 10E presents the expression changes of twelve genes from the risk model as they pertain to the transition process from epithelial cells to squamous differentiated cells. Functional enrichment analysis of differentially expressed genes between squamous differentiated cells and epithelial cells revealed enrichment in “peptidyl-lysine modification”, “protein acetylation” (an epigenetic modification sharing enzymes with lactylation modification), and “histone modification” (Figure 10F). These results suggest the significant role of lactylation modification in the process of epithelial-to-squamous cell carcinoma transition in bladder cancer.

### 3.9. Spatial Transcriptome Analysis in BLAC

**Figure 11:**
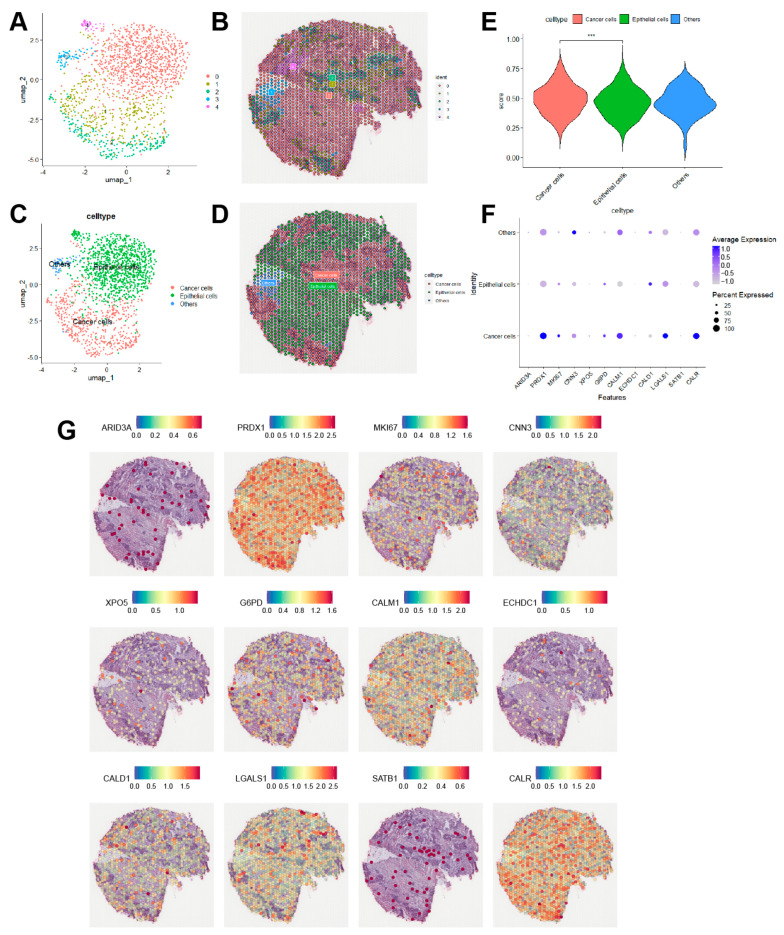
Spatial transcriptome analysis in BLAC. (**A**,**B**) Dimensionality reduction Umap plot of spatial transcriptomics data and spatial distribution map of each cluster. (**C**,**D**) Umap plot and spatial distribution of each cell type after annotation. (**E**) Scores of model for each cell group. *** *p* < 0.001. (**F**) Bubble plot indicated that 12 model genes were significantly higher in cancer cells than in epithelial cells. (**G**) Spatial distribution of 12 model genes.

Spatial transcriptomics enables researchers to explore gene expression profiles in relation to spatial characteristics. Publicly available spatial transcriptomics data from muscle-invasive BLAC were processed using the “Seurat” package and dimensionally reduced via the umap method (Figure 11A,B). Figure 11C,D illustrates the cellular clustering derived from cell annotation and their corresponding spatial distribution characteristics. Upon assigning scores to cell populations using the risk model, it is evident that cancer cells exhibit significantly higher scores compared to epithelial cells (Figure 11E), which corroborates the findings from single-cell analysis. Bubble plots further indicate that twelve model genes are highly expressed in tumor cells (Figure 11F). Lastly, Figure 11G presents the spatial expression patterns of these model genes across the tissue section.

## 4. Discussion

Lactate is an important metabolite. It was once regarded as a byproduct of an organism’s ability to maintain its energy supply in the absence of oxygen. However, the discovery of lactylation modification in 2019 has greatly overturned people’s understanding of it. With the continuous advancement of research, lactylation modification has been proved to play a crucial role in many fields. In various cancers, lactylation has been shown to be an important way for tumor cells to modify the tumor microenvironment and exacerbate their malignant behavior [27,28,29,30,31,32]. However, in the field of BLAC, although researchers have found that lactylation modification is significant for the occurrence and development of BLAC [10,21,22], no prognostic model based on this biological process has been proposed. This study addresses this knowledge gap in the field of BLAC research.

In this study, a prognostic model of BLAC based on lactylation modification genes was established. Twelve genes were identified in this prognostic model: ARID3A, PRDX1, MKI67, CNN3, XPO5, G6PD, CALM1, ECHDC1, CALD1, LGALS1, SATB1, and CALR. ARID3A encodes a DNA-binding protein. ARID3A promotes tumor growth by interacting with CEP131 to promote the expression of stem cell-like genes [33]. PRDX1 encodes a peroxidase. Celastrol upregulates the reactive oxygen species (ROS) level and promotes apoptosis in colorectal cancer (CRC) cells by inhibiting the antioxidant activity of PRDX1 [34]. MKI67 encodes an important marker protein for tumor proliferation activity. CNN3 encodes a calcium regulatory protein that acts as a potential oncogene in cervical cancer [35]. Exportin-5 (XPO5) encodes a protein responsible for the delivery of precursor mirnas out of the nucleus. A member of the nucleoprotein family, it is a key protein responsible for the transport of precursor mirnas from the nucleus to the cytoplasm. In a cohort of colorectal cancer patients, high XPO5 expression was associated with lower survival and worse pathological features [36]. G6PD encodes a key enzyme mediating the pentose phosphate pathway. In cervical cancer, the lactylation of DCBLD1 stabilizes its conformation and inhibits the degradation of G6PD, which in turn upregulates the pentose phosphate pathway within tumors and promotes cervical cancer progression [37]. In addition, HPV16 E6 can promote G6PD activity by inhibiting its lactylation, which also leads to upregulation of the pentose phosphate pathway and promotes the progression of cervical cancer [38]. CALM1 encodes a calcium-binding protein that is associated with esophageal squamous cell carcinoma and resistance to EGFR-antagonistic muscle [39]. ECHDC1 encodes an enoyl-CoA hydratase. A multi-modal meta-analysis has suggested that ECHDC1 can be used as a new breast tumor suppressor [40]. Silencing ECHDC1 in bladder cancer cell lines induces upregulation of p27, ultimately leading to cell cycle arrest and reduced proliferation [41]. CALD1 encodes a calmodulin-binding protein, which is closely related to tumor-associated fibroblasts in BLAC and significantly affects the progression and prognosis of BLAC [42]. LGALS1 encodes galectin-1. This protein can promote the occurrence and development of BLAC through the JNK pathway [43]. SATB1 encodes a multipotent nuclear matrix-binding protein, which can promote the development of prostate cancer by regulating the epithelial–mesenchymal transition [44]. CALR encodes mainly involved in protein folding and quality control and it is closely related to myeloproliferative neoplasms [45]. In summary, the twelve model genes not only have sites for lactylation modification [24], but also have an inseparable relationship with the occurrence and development of tumors. In addition, there are few studies on the above-mentioned genes in bladder cancer and lactylation modification. The results in this study suggest the potential of these genes as research targets for lactylation modification in bladder cancer.

Based on the aforementioned model, an independent prognostic analysis was first conducted. Univariate and multivariate Cox analyses demonstrated that the risk scores derived from the model could serve as independent risk factors for bladder cancer in both the training and validation cohorts. Next, an analysis of the immune microenvironment was performed. On the one hand, the high-risk group exhibited heightened immune suppression: in terms of immune cell infiltration, there was increased infiltration of regulatory T cells and suppressed dendritic cells, alongside decreased NK cell infiltration; in terms of immune function, a significant increase in the co-inhibition of antigen-presenting cells and T cells was observed. However, on the other hand, immune activation was also upregulated in the high-risk group. Due to the limited interpretative capacity of transcriptomic data regarding immune status, we speculate that this result may be due to immune heterogeneity among patients. However, a more detailed explanation relies on further research. Although the immune infiltration algorithm provides a comprehensive description of the immune infiltration status in patients, the conclusions drawn in this section still require experimental validation. Lactylation has been shown to suppress the immune activity of macrophages [46,47] and CD8+ T cells [48]. Therefore, co-culturing tumor cells with immune cells from different immune score groups could further validate the conclusions presented in this study. Tumor microenvironment analysis showed that the high-risk group had lower tumor purity, which is consistent with previous reports that lower purity in bladder cancer is associated with poorer prognosis [49,50]. Subsequent analysis of treatment responsiveness indicated that the high-risk group was more sensitive to most first-line chemotherapeutic drugs for bladder cancer, but showed poorer efficacy with some targeted therapies such as erlotinib. It should be noted that due to algorithmic limitations, we used IC50 to represent drug sensitivity. However, IC50/EC50 is a better representation of drug sensitivity. Although patients in the high-risk group theoretically exhibit greater sensitivity to chemotherapy, validation through in vitro and in vivo experiments is particularly crucial. In this study, the high-risk group exhibits higher glycolytic activity, suggesting an elevated level of lactylation. High lactylation levels in bladder cancer have been shown to confer resistance to cisplatin-based chemotherapy [21]. Furthermore, the upregulation of lactylation on NBS1 has been associated with enhanced resistance to chemotherapy-induced DNA damage [51]. This is consistent with the findings of our study. Future research could further validate this issue. TIDE analysis suggested that the high-risk group had a lower response rate to immunotherapy. Considering the results from enrichment analysis, which showed significant upregulation of hypoxia and glycolysis pathways in the high-risk group, it is believed that the elevated glycolytic metabolism in the high-risk group leads to increased environmental lactate concentration. The elevated environmental lactate concentration raises the level of protein lactylation modifications in infiltrating cells, ultimately creating an immunosuppressive microenvironment, resulting in a poorer response to immunotherapy.

Multi-omics analysis is a promising method for advanced analysis. The advantage of using different omics data allows researchers to analyze biological processes from multiple perspectives. In this paper, based on the use of bulk RNA-seq data, single-cell transcriptome data and spatial transcriptome data were used to further explore the biological significance of prognostic models. In the results presented here, GO analysis of 75 genes related to lactylation modification revealed extensive enrichment in the cell–matrix junction structure and the connection of various cytoskeletal proteins. GO analysis of differentially expressed genes in the high- and low-risk groups revealed enrichment in extracellular matrix construction and degradation. Interestingly, fibroblasts obtained the highest lactylation-related model score in single-cell sequencing. It has been reported that the expression of Glucose transporter 1 (Glut1) in tumor-associated fibroblasts of hepatocellular carcinoma (HCC) is upregulated, promoting the glycolytic pathway [52]. In oral tongue squamous cell carcinoma, clinical data prove that fibroblasts are positively correlated with the glycolysis level of the tumor microenvironment [53]. Cancer-associated fibroblasts in pancreatic cancer reutilize lactate produced by tumor cells through monocarboxylate transporter 1 to promote their own proliferation and create a fibrotic tumor microenvironment [54]. In addition, fibroblasts can also elevate the glycolysis level of head and neck squamous cell carcinoma by secreting hepatocyte growth factor (HGF). In conclusion, the results presented here and existing literature reports together demonstrate a complex and tight link between lactate, lactylation modification, and fibroblasts. On one hand, the fibroblasts in the tumor microenvironment may promote the enrichment of lactate in the microenvironment by increasing the glycolysis level, thereby elevating the level of cellular protein lactylation modification in the microenvironment. On the other hand, the increased level of lactylation modification is likely to modify the biological behavior of fibroblasts, facilitating tumor cells to accomplish the remodeling of the microenvironment matrix. Additionally, this study used pseudotime analysis of single-cell sequencing to demonstrate the dynamic changes of the 12 model genes during the transition of bladder epithelial cells to squamous cell carcinoma. Our analysis highlights the correlation between risk scores and the transition to squamous cells, underscoring a key aspect of bladder cancer progression. While our findings are compelling, we urge the research community to exercise caution regarding these results until further validation is conducted. Lactylation has been shown to be significantly upregulated in various cancers, including breast cancer [55], liver cancer [56], colorectal cancer [20], and glioma [28]. Our single-cell results indicate an elevation in glycolytic activity during the development of bladder squamous cell carcinoma, suggesting that the intracellular level of lactylation may also be increased during this process. Future studies could further validate this hypothesis using the SCaBER cell line, a bladder squamous cell carcinoma cell line. More importantly, GO analysis indicated that “peptidyl-lysine modification”, “protein acetylation” and “histone modification” are the significantly enriched biological pathways for the differential genes between these two cell types. Firstly, all known lactylation modifications occur on lysine residues [57]. Secondly, the GO database currently lacks pathways specifically labeled as “lactylation modification”, while acetylation modification is a post-translational modification highly similar to lactylation. These two modifications share many writer and eraser enzymes [6,23]. This raises an important question regarding the role of the interaction between acetylation and lactylation modifications in the epithelialization of bladder cancer. Lactylation and acetylation share many common histone modification sites, such as H3K18, H3K27, and H3K23 [6]. In normal cells, the concentration of lactyl-CoA is approximately 1/1000th that of acetyl-CoA [58], suggesting that lactylation has a limited competitive capacity in the context of the intense competition between the two modifications. Understanding how lactylation competes with acetylation during the development of bladder squamous cell carcinoma is an intriguing question. Lastly, histone modification is one of the most important ways lactylation modification regulates the cellular metabolic network [59]. In summary, the results of this study suggest a probable presence of significant lactylation modifications during the transition of bladder epithelium to squamous carcinoma. Furthermore, both single-cell analysis and spatial transcriptome analysis indicated a significant enhancement in the model scores of epithelial cells after carcinogenesis, as anticipated.

To enhance the clinical applicability of our scoring system, it is crucial to validate its effectiveness in external BLAC patient cohorts. Prospective studies will enable us to collect data during actual treatment processes, allowing for further assessment of the association between treatment response and survival rates. This not only provides a reference for clinicians but also offers better treatment options for patients. Additionally, future research utilizing immunoprecipitation and mass spectrometry can delve into the specific sites of lactylation and their roles in tumor biology. Such detailed analyses will help elucidate how lactylation affects protein function and its potential impact on tumor behavior, thereby providing new insights for future targeted therapeutic strategies.

In conclusion, this study reported a novel prognostic model of lactylation modification in BLAC. This model possesses satisfactory predictive power and holds the potential to guide clinical treatment. In addition, the model has significant biological implications at both the single-cell level and the spatial level. However, this study still has certain limitations: as the research in the field of lactylation modification is still in the preliminary stage of exploration, how the 12 model genes affect tumors through lactylation modification remains an open issue. In addition, the results obtained in this paper still need to be verified by larger scale clinical data to achieve a better translational effect. Nevertheless, we believe that these issues will be addressed in the near future as researchers increasingly focus on and understand the modification of lactylation.

## Data Availability

The datasets used in this study can be found online as described above. The TCGA database is located at https://portal.gdc.cancer.gov (accessed on 5 November 2024); the GEO database is located at https://www.ncbi.nlm.nih.gov/gds (accessed on 5 November 2024).
